# How Internal Political Efficacy Translates Political Knowledge Into Political Participation: Evidence From Germany

**DOI:** 10.5964/ejop.v12i2.1095

**Published:** 2016-05-31

**Authors:** Frank Reichert

**Affiliations:** a Faculty of Education and Social Work, University of Sydney, Sydney, Australia; Aalborg University, Aalborg, Denmark

**Keywords:** action theory, Germany, internal political efficacy, political knowledge, political participation, Theory of Planned Behaviour, voting

## Abstract

This study presents evidence for the mediation effect of political knowledge through political self-efficacy (i.e. internal political efficacy) in the prediction of political participation. It employs an action theoretic approach—by and large grounded on the Theory of Planned Behaviour—and uses data from the German Longitudinal Election Study to examine whether political knowledge has distinct direct effects on voting, conventional, and/or unconventional political participation. It argues that political knowledge raises internal political efficacy and thereby indirectly increases the chance that a citizen will participate in politics. The results of mediated multiple regression analyses yield evidence that political knowledge indeed translates into internal political efficacy, thus it affects political participation of various kinds indirectly. However, internal political efficacy and intentions to participate politically yield simultaneous direct effects only on conventional political participation. Sequentially mediated effects appear for voting and conventional political participation, with political knowledge being mediated by internal political efficacy and subsequently also by behavioural intentions. The mediation patterns for unconventional political participation are less clear though. The discussion accounts for restrictions of this study and points to questions for answer by future research.

It is commonly understood that people who are more knowledgeable in political realm and who feel more efficacious to influence political decisions are more politically active. Although political knowledge may be considered a significant quality of politically active and involved citizens, most people’s knowledge about politics appears insufficient to meet the standards of a “competent citizen” (Delli Carpini & Keeter, 1996; Maier, 2000). Moreover, there is still a need to understand how political knowledge might be relevant to increasing political participation. On the one hand, action theory suggests that subjective feelings of political efficacy might be more relevant than factual knowledge when predicting actual behaviour. On the other hand, the aforementioned scholars also suggest that political knowledge be a vital determinant of political efficacy. Therefore, in order better to understand the *mechanism* of how political knowledge might influence citizens’ participation in the political realm, this article examines internal political efficacy as a potential mediator of political knowledge: Following action theory, it argues that political knowledge stimulates the individual’s sense of political efficacy, which operates as a mediator by transmitting a substantial amount of that knowledge’s impact on actual participation in politics. The major contribution of this analysis is the application of action theory to the political realm using representative panel data, thereby going beyond previous research in the field. The present study focuses on Germany, where longitudinal data are available, and thus mainly research from a German context is reflected; acknowledging that the predictors of participation may vary across national contexts.

## Political Knowledge, Internal Political Efficacy and Political Participation

### Political Knowledge

Political knowledge is “the range of factual information about politics that is stored in long-term memory” (Delli Carpini & Keeter, 1996, p. 10). More extensive knowledge about polity, politics, and policy is presumed to enable and encourage people to participate in politics (Delli Carpini & Keeter, 1996; Galston, 2001). Indeed, empirical evidence suggests that political knowledge contributes to more stable and consistent political attitudes, helps citizens achieve their own interests and make decisions that conform with their attitudes and preferences, promotes support for democratic values, facilitates trust in the political system, and motivates political participation (Galston, 2001). It is therefore not merely rational to acquire political knowledge for self-interest purposes, but political knowledge is also a motivational force. As such a multifunctional variable, it increases the chance *effectively* to achieve own political goals. The mechanism how political knowledge translates into political behaviour is not fully understood yet, however, but it is sensible to assume that this mechanism might involve an individual’s political self-efficacy (see below).

### Internal Political Efficacy

The concept of “self-efficacy” relies on the distinction between “outcome expectations”—“a person’s estimate that a given behavior will lead to certain outcomes”—and “efficacy expectations”—“the conviction that one can successfully execute the behavior required to produce the outcomes” (Bandura, 1977, p. 193). The latter is an individual’s expectation of being able to act successfully to gain the objective aimed. With regard to politics, this comprises the *feeling* that one is capable of understanding political facts and processes and that one feels capable of influencing politics successfully (Almond & Verba, 1965; Balch, 1974; Campbell, Gurin, & Miller, 1954). Consequently, internal political efficacy involves a person’s subjective assessment of his or her political knowledge, among other things such as the belief in one’s behavioural skills and that the actions undertaken will have an impact. Although some scholars use different vocabulary, such as an individual’s “self-concept of political competence” (Krampen, 2000b), “political self-efficacy” (Caprara, Vecchione, Capanna, & Mebane, 2009; see also Madsen, 1987, whose construct of “political self-efficacy” joins items that reflect both internal and external efficacy) or “subjective political competence” (Koch, 1993; Pickel, 2002; Reichert, 2013), these concepts equate to internal political efficacy, which is the more common term. That is, the self-concept of an efficacious citizen describes him or her as politically competent and powerful. It is therefore no surprise that internal political efficacy has been considered as a motivational factor in rational pathways to political participation (Finkel, 1985; see also Jung, Kim, & de Zúñiga, 2011), as it combines both elements of rationality and motivation.

Indeed, numerous studies show that internal political efficacy plays a significant, positive role in political participation (Condon & Holleque, 2013; Krampen, 2000b; Schulz, Ainley, Fraillon, Kerr, & Losito, 2010). Krampen (2000b, p. 290) concludes, for instance, “the variables of frequency of political activity in everyday life, self-concept of political competence, and political knowledge in adolescence are the most significant discriminators for voting versus nonvoting behavior of young adults.” Hence, both political knowledge and internal political efficacy are precursors of political participation, and some scholars suggest that this might also hold for political activities other than voting (Gallego & Oberski, 2012; Johann, 2012; Westle, 2011, 2012).

### Kinds of Political Participation

When we talk about “political participation”, we refer to activities undertaken voluntarily by a citizen to influence authoritative or generally binding regulations and decisions related to the political system (van Deth, 2014). Behavioural intentions are strong predictors of actual behaviour (Ajzen, 2012), although the correlational relationship between both is by no means perfect (Fishbein & Ajzen, 1981; Gaiser & de Rijke, 2010). Even so citizens’ intentions to perform political behaviour are often conceptualised as political participation too.

We may distinguish four kinds of political participation (e.g., Barnes et al., 1979; Steinbrecher, 2009): *Voting* in political elections does not require intense effort, nor is it bound to a strong commitment. The only constraint on voting is formal regulations (e.g., citizenship). *Conventional* political activities are traditional, often institutionalized and *party-related* forms of participation (e.g., supporting an election campaign). These activities ask for a higher degree of commitment and a more extensive investment of time by its activists. *Unconventional* activities refer to a broad range of less institutionalized and usually less time-intensive or committed political participation located outside political parties (e.g., attending a non-violent political protest march) that often deal with rather narrow social or political issues or aim at solving a certain political problem. Alternatively, this kind of participation could be labelled “issue-based participation”, even though the present research employs the more traditional terminology. *Non-normative* political activities, such as participation in an unauthorised or violent political demonstration, are located outside the legal framework and will not be considered here.

### Political Knowledge, Efficacy and Participation

It is obvious that political knowledge, internal political efficacy and political participation must be correlated with each other, and research suggests that political knowledge and efficacy are of greater predictive value for explaining political participation than vice versa (Finkel, 1985; Vetter & Maier, 2005). According to contemporary wisdom, political knowledge “promotes political participation” (Galston, 2001, p. 224), and the same applies to internal political efficacy, as many studies report a positive impact of political efficacy on a range of political activities (Balch, 1974; Steinbrecher, 2009; Vecchione et al., 2014; Verba, Schlozman, & Brady, 1995). While much empirical research was concerned with voting behaviour, showing that political knowledge increases the likelihood to vote (e.g., Amadeo, Torney-Purta, Lehmann, Husfeldt, & Nikolova, 2002; Delli Carpini & Keeter, 1996; Howe, 2006; Krampen, 2000b), research has also focused on other kinds of political participation, however. On the one hand, scholars find the influence of political knowledge to be smaller with respect to other political activities (Milner, 2007; Oesterreich, 2003), and others even suggest that internal political efficacy might be the more robust predictor of unconventional political activities in comparison to the impact of political knowledge (Kuhn, 2006; Schulz et al., 2010). On the other hand, some studies provide evidence for the simultaneous predictive value of internal political efficacy and political knowledge on institutionalized *and* unconventional political activities (Gallego & Oberski, 2012; Johann, 2012; Westle, 2011, 2012). Hence, there is a need better to understand the mechanism of how political knowledge and efficacy affect political participation, because most previous research was constrained to the examination of bivariate relationships or models that included multiple predictors of participation, without focusing on the mechanism behind political knowledge and efficacy.

## Towards an Action Theoretic Model of Political Participation

Evidence for the role of internal political efficacy versus political knowledge with respect to political participation is equivocal—especially because positive correlations between objective and subjective measures are only of moderate extent, at least in the German context, as shown for different measures of objective and subjective political knowledge (Maier, 2000; Oberle, 2012; Westle, 2009). This might support the assumption that both political knowledge and internal political efficacy have separate effects on political participation. On the other hand, action theoretic models of behaviour ask *how* political knowledge translates into political participation and whether its potential effect is direct, indirect, or both direct and indirect.

Specifically, action theoretic models argue that it is about personal convictions of control and feelings of self-efficacy, yet not actual knowledge and skills that are most important in *initiating* behaviour (Ajzen, 1991; Bandura, 1977; Krampen, 2000a). The Theory of Planned Behaviour (TPB) (Ajzen, 1991), in particular, shows that (normative, behavioural and control) beliefs determine behaviour. Conceptually, perceived behavioural control corresponds to self-efficacy, which refers to internal political efficacy in political realm. Moreover, research conducted in the vein of TPB suggests that accurate knowledge of a certain topic is neither sufficient nor necessary to predict actual behaviour (Ajzen, Joyce, Sheikh, & Cote, 2011). One study that employed TPB, for instance, found that overall blood donation knowledge did not predict blood donation intention directly, but it had the strongest mediation effect through self-efficacy (Polonsky, Renzaho, Ferdous, & McQuilten, 2013).

Similarly, a study on the willingness to communicate about an environmental issue yielded positive effects of perceived knowledge about risks related to that issue on self-efficacy (Dalrymple, Shaw, & Brossard, 2013). While perceived knowledge had no significant effect on the willingness to communicate, self-efficacy had significant positive effects. Another study on the smoking behaviour of youths yielded that adolescents’ perceptions of their parents’ knowledge about the offspring’s whereabouts and activities reduced the adolescent’s intention to smoke and actual smoking behaviour indirectly through self-efficacy not to smoke (Harakeh, Scholte, Vermulst, de Vries, & Engels, 2004).

In contrast, among the few studies in the tradition of TPB that are loosely related to forms of socio-political participation, some modelled a direct effect of knowledge on blood donation intentions or on wastepaper recycling behaviour and yielded rather insignificant or mixed results (Cheung, Chan, & Wong, 1999; Lemmens et al., 2005). Hence, only internal political efficacy might be a direct predictor of political participation, as it could fully mediate the effect of political knowledge. Following these thoughts, maybe subjective control and *perceptions* of efficacy are stronger predictors of political behaviour than actual knowledge about politics, and internal political efficacy might work as a mediating mechanism?

Such a mediation effect of political efficacy was, for example, discussed by Delli Carpini and Keeter (1996); and Jung et al. (2011) modelled an effect of political knowledge on internal political efficacy, while both were supposed to promote political participation. Although these scholars did not statistically test this mediation effect, they implicitly assumed—and their results suggest the thesis—that political efficacy mediates the facilitating effect of political knowledge. Conversely, Vetter and Maier (2005) find the regression coefficients in regression analyses to be slightly stronger for internal political efficacy predicting political knowledge than vice versa, though these scholars also did not test for mediated relationships. Multiple mediation analyses of a study which was conducted in Germany provide some evidence that internal political efficacy might indeed be the more proximal measure, as it mediated the effect of political knowledge on actual as well as intended conventional political participation (Reichert, 2013). Due to limitations with respect to the sample, however, that study did not find mediated or direct effects of political knowledge with regard to other forms of political participation. In particular, the analysis by Reichert (2013) had the character of a pre-test and exclusively comprised 76 university students of psychology. Furthermore, only 41 students participated twice, and since no federal election took place during that study period, an examination of actual voting behaviour was impossible. Hence, that research needs to be expanded in scope, owing to its obvious limitations, to examine whether the proposed mediation mechanism holds for the entire population.

Finally, we also need to address the role of behavioural intentions. TPB does not only posit that internal efficacy affects actual behaviour, but that it also predicts behavioural intentions which additionally mediate efficacy’s impact on actual behaviour, while efficacy retains its direct effect—above and beyond the significant impact of behavioural intentions (Ajzen, 1991, 2012). Based on recent research which indicates that internal political efficacy is of special significance in the prediction of conventional political participation (Reichert, 2013), it is also necessary to examine whether internal political efficacy outweighs the impact of behavioural intentions with regard to conventional participation.

In conclusion, the contribution of the present study lies in the examination of the mechanism how political knowledge affects different kinds of political participation, and whether its influence is mediated by internal political efficacy. Furthermore, this study analyses a sequential mediation with behavioural intentions as additional mediators. Although some scholars have discussed a mediated effect of political knowledge (Delli Carpini & Keeter, 1996; Jung et al., 2011), this mechanism has not yet been tested in the political realm, except for one study with severe limitations (Reichert, 2013). In comparison, a major feature of the present analysis is the utilisation of panel data, which enable the examination of the suggested mediation mechanism with respect to actual (instead of merely intended) political behaviour using a more representative and comprehensive sample.

## Hypotheses

The research question that is central to this analysis asks how political knowledge influences different kinds of normative political participation, and it aims to clarify the role of internal political efficacy as a mediating mechanism. Such a mechanism has been proposed by other scholars without proper testing (Delli Carpini & Keeter, 1996; Reichert, 2013). Following those suggestions and TPB, we expect indirect (positive) influences of political knowledge on political participation, mediated by internal political efficacy due to an overlap of both variables in multiple regression analyses (H1). In particular, we assume the effect of political knowledge is fully mediated by internal political efficacy with regard to conventional political participation, i.e. political knowledge will not retain any effect on conventional participation when we control for political efficacy (H2). Referring to the study by Reichert (2013), which found no mediated effects for other forms of political participation, however, it needs to be explored whether and to what extent this mechanism also applies to voting and unconventional political participation. Additionally, the impact of internal political efficacy may be mediated by behavioural intentions—which implies a sequential mediation of political knowledge by both internal political efficacy *and* willingness to participate (H3). This assumption derives directly from TPB which claims that both efficacy *and* behavioural intentions directly affect actual behaviour, while efficacy is additionally mediated by behavioural intentions. Although the strength of this impact of political efficacy may vary across different forms of political participation, we expect them to be strongest—and more important than behavioural intentions—with regard to conventional political participation, based on study findings according to which particularly internal political efficacy and conventional political participation are positively correlated (Reichert, 2013) (H4).

## Method

### Procedure

For the purpose of this study, we utilise the German Longitudinal Election Study (GLES) long-term panel (Rattinger et al., 2012). We focus on Germany as this is the home of the researcher, and it is the context where a previous study tapped into this mediation relationship, yet that study had obvious limitations with respect to its sample in terms of size, representativeness and the time period covered (Reichert, 2013). The GLES provides an opportunity to test the introduced hypotheses, as this dataset includes measures for political knowledge, internal political efficacy, and information about all three kinds of intended as well as actual political participation across time, which is quite rare. Hence, the GLES long-term panel is an appropriate choice, despite some constraints (see below).

The GLES long-term panel merges data from pre- and post-election surveys conducted around the elections to the German *Bundestag* in 2002, 2005, and 2009 using a disproportionate stratified multistage random sampling design. The population includes people with German citizenship aged 16 years and older who lived in Germany at the time of measurement. Of 3,263 people surveyed in 2002, 902 participated again in the second wave in 2005, and still 641 participated in the third wave in 2009.^i^ Because of the disproportionate sample design, the east/west weight was employed in all analyses. Cases with “mutations” in age or gender were excluded. Similarly, participants with a reported immigration background were also eliminated from the analysis, due to the low number in the sample in comparison to the true population proportion (those who refused to report whether they were born in Germany were excluded too).

### Measures

The independent variables used in this study were measured in 2002, while the dependent variables—political participation—were measured in 2005 and in 2009.^ii^ The means and standard deviations of all independent and dependent variables are depicted in Table A1 (Appendix).

#### Political Knowledge and Efficacy

##### Political Knowledge

Two questions in the 2002 survey tapped political knowledge explicitly: “Which of the two votes in the federal election is of more importance?” and, “How many federal states does Germany have today?” Both items are relevant in the context of elections and parliaments: The first item refers to the second vote (of two votes) in federal elections which decides about the allocation of seats in the federal parliament, the German *Bundestag*, and thus determines which political party will be given the task of forming a government. The second item is of significance with respect to the German *Bundesrat*, a constitutional body which represents all German federal states (*Länder*) at the national level. The fact that 16 federal states exist is political knowledge as the *Länder* are represented in the *Bundesrat* with respect to their size and population.

Furthermore, participants were asked about the political stances of several nationwide important political parties on the political issues of nuclear energy, the immigration of foreigners, and the European unification. For each item and political party, they responded on a seven-point scale, each of which was labelled with opposing statements at the extremes; for example, “laws on immigration should be relaxed” versus “laws on immigration should be made tougher,” to indicate how determined or close to one of these extremes they believed a political party’s position was. We additionally utilized these data, that is, what respondents thought the positions of the political parties were on those political issues, since two items seemed to be inadequate in the measurement of political knowledge. Consequently, all items related to political elections, and thus measured only certain aspects of political knowledge.

In particular, the respective election manifestos of each party were the basis for evaluating the “correct” party position on the aforementioned three political issues and for deciding whether respondents’ knowledge about the stances of six German political parties on those issues was correct (i.e. eighteen additional political knowledge items). This seemed reasonable, because the GLES was always conducted around federal elections, and manifestos are fixed information that voters can read to learn about the positions of political parties. Two coders indicated which scale point most adequately represented the respective position of each party, referring to their election manifestos. After all items had been coded independently by each coder, both coders discussed all codings, and in particular diverging codings—most discrepancies were only of a difference of one scale point, and for every item they came to an agreement about the specific scale point that best reflected the “correct” response (the overall inter-coder reliability is .85).

For all 20 knowledge items, “don’t know” was treated as missing data, because research suggests that missing knowledge (“don’t know”) differs from incorrect knowledge (e.g., Johann, 2008; Mondak, 1999). In particular, internal political efficacy correlates with nonresponse to the political knowledge questions (Mondak, 1999), as was the case in the present sample (*r* = -.35).^iii^ Eventually, this resulted in an additive political knowledge index representing the percentage of correct answers.

##### Internal Political Efficacy

Three efficacy items were used as indicators for internal political efficacy (0 = *strongly disagree* … 4 = *strongly agree*):^iv^ “Politics is such a complex issue that people like me cannot understand what is going on” (reverse coding); “I feel I could play an active role in a group dealing with political issues;” and, “I am perfectly able to understand and assess important political questions.” A mean index was calculated (Cronbach’s α = .70), which correlated significantly with political knowledge (*r* = .12, *p* < .001).

#### Political Participation

With respect to political participation, post-election participants answered whether they had voted in the recent federal election (0 = *no* vs. 1 = *yes*), whereas pre-election participants indicated on a five-point scale how likely it was that they would vote in the forthcoming federal election (0 = *I will certainly not vote* … 4 = *I will certainly vote*). Self-reported, retrospective information about voting decisions in the elections of the previous period of the German *Bundestag* were always available (recoded to 0 = *did not vote* vs. 1 = *voted*).

Two items related to actual conventional political participation: “I visited election campaign rallies” (five-point scale recoded to 0 = *never* and 1 = *rarely* to *very often*; not available in 2005), and membership in a political party (0 = *not a member* and 1 = *member*). The final index was coded *no participation at all* (0) and *participation in at least one activity* (1). “Try to get support from a party/politician” (0 = *I would definitely not do* … 4 = *I would definitely do*) measured the intention to participate in conventional political participation.

The measurement of unconventional participation was more difficult, since only a single item was available, measuring “Membership in a citizen’s initiative.” Consequently, this measure reflects a specific form of unconventional or issue-related political participation. For willingness to participate unconventionally, however, two items could be used: “Contribute to a citizens’ initiative,” and, “Participate in a legal demonstration” (0 = *I would definitely not do* … 4 = *I would definitely do*).

#### Socio-Demographic Control Variables

All analyses controlled for potential confounders. Specifically, standard socio-demographic variables that usually correlate with political knowledge, efficacy or/and participation were used (Delli Carpini & Keeter, 1996; Grönlund & Milner, 2006; Maier, 2000; Stolle & Gidengil, 2010; Vetter, 2006; Westle, 2012).^v^ The list of control variables comprised gender (0 = *female* vs. 1 = *male*; 51% male^vi^), age (*M* = 50.85, *SD* = 18.05), and a household’s total net monthly income, which was recoded into eight nearly equally spaced intervals ranging from *less than 500 Euro* to *at least 3,500 Euro* (*M* = 3.27, *SD* = 2.04, i.e an average income of approximately 1885 Euro). For educational achievement, information about the highest school graduation certificate was recoded into two dummy variables “no or lowest formal qualification” (46%), and “intermediary secondary qualification” (30%; remainders with higher qualification) (0 = *no* vs. 1 = *yes*). Dummy variables for region of residence (0 = *West Germany* and 1 = *East Germany*; 77% located in West Germany) as well as for *pre-* (0) vs. *post-election* date of interview (1; 52% pre-election) were also considered as control variables, because differential political socialization and knowledge bases between Eastern and Western Germans may be substantial. Moreover, people tend to have higher levels of political knowledge after political elections compared to before political elections (Maier, 2009; Westle, 2012), although election campaigns do not seem to reduce the knowledge gap (McCann & Lawson, 2006).

## Results

### Data Analysis

Multiple analyses accounted for political knowledge, internal political efficacy and the control variables mentioned above as predictors, including behavioural intentions as sequential mediators (except for voting in the post-election sample). Longitudinal logistic regression analyses were performed for actual participation between 2002 and the measurement of the criteria. Linear regression was employed to predict the mediators, because these variables approached an interval scale. Analyses were conducted in *Mplus* (Muthén & Muthén, 1998-2015) using its integrated full information maximum likelihood procedure for treating missing data in the dependent variables, and employing a robust maximum likelihood estimator and Monte Carlo integration with 750 integration points for sequential mediation analyses. For continuous criteria, the common coefficient of determination is provided in the text (*R*^2^), whereas for binary dependent variables, Nagelkerke’s adjusted coefficient of determination (*Ṟ*^2^) was calculated manually (Nagelkerke, 1991, p. 692, equation (3)).

### Explaining Political Participation

Before we proceed with the results of the multiple regression and mediation analyses, it is noteworthy that all correlations between actual participation or intended participation and political knowledge and internal political efficacy were positive (Table A2 in Appendix). Among the post-election sample, casting one’s ballot correlated marginally significantly with political knowledge but not with political efficacy. Furthermore, internal political efficacy was a significant correlate of all other behavioural variables, whereas political knowledge was a significant correlate of behavioural intentions only (though marginally significant for intentions to vote), yet knowledge did not correlate significantly with either kind of actual participation. Finally, almost all correlations between our criteria and internal political efficacy were stronger than those between participation and knowledge. It therefore seems plausible to expect mediated relationships between political knowledge and actual political participation, as these preliminary analyses reinforce the expectation that political knowledge is mediated by internal political efficacy (Delli Carpini & Keeter, 1996; Reichert, 2013) and not vice versa (as indicated by Vetter & Maier, 2005). That is, a significant zero-order correlation between predictor and outcome variable is not an essential requirement for a mediated relationship (MacKinnon, Fairchild, & Fritz, 2007), in particular if the mediation process becomes less proximal as may be the case in longitudinal analyses with several years between the measurement of the independent and the dependent variables (Shrout & Bolger, 2002).

#### Examining Internal Political Efficacy as Sole Mediator

In Table 1, we see that political knowledge did not significantly predict actual political participation in any model, i.e. accounting for other influences, a citizen’s knowledge about politics did not explain his or her political behaviour. More interestingly, internal political efficacy was irrelevant for voting, though efficacy significantly increased the chance to engage in conventional political participation. Since both political knowledge and efficacy were significantly and positively correlated, internal political efficacy mediated the effect of political knowledge on political participation with regard to conventional political behaviour, i.e. political knowledge contributed to internal political efficacy, and its influence on conventional political participation (as well as its effect on unconventional political behaviour in 2005) was transmitted via a citizen’s political self-efficacy. Since this pattern of correlations did not appear for all forms of political participation, other predictors might have been at work for those activities.

**Table 1:** Longitudinal Mediated Regression Analyses (Dependent Variables: Political Participation)

	Voting			Conventional Participation^c^		Unconventional Participation^d^	
Predictor	2002^a^	2005^a^	2009^b^	2005	2009	2005	2009
Political knowledge	2.06 (5.72)	-5.87 (3.79)	2.91 (2.24)	0.68 (1.13)	-0.11 (0.99)	-0.52 (1.73)	0.47 (1.11)
Internal political efficacy	0.46 (0.49)	-0.37 (0.60)	-0.01 (0.37)	0.69** (0.21)	0.90*** (0.19)	0.54* (0.24)	0.19 (0.34)
Political action (behavioural intention)	1.84*** (0.31)	1.66** (0.53)		0.32** (0.12)	0.29** (0.10)	0.36 (0.22)	0.64^†^ (0.35)
Age	+0.00 (0.03)	0.10* (0.05)	0.03 (0.02)	0.04*** (0.01)	0.02* (0.01)	0.02 (0.02)	0.01 (0.02)
Gender	0.21 (0.78)	0.86 (1.08)	0.62 (0.64)	0.01 (0.28)	-0.28 (0.27)	-0.01 (0.44)	1.14 (0.73)
Pre-/Post-election				-0.13 (0.26)	0.77** (0.26)	0.15 (0.44)	-0.16 (0.50)
Income	0.11 (0.22)	0.42* (0.19)	0.07 (0.18)	0.05 (0.07)	0.01 (0.07)	-0.02 (0.10)	-0.06 (0.13)
Region of residence	1.30 (1.20)	1.21 (3.20)	-1.81** (0.57)	-1.05* (0.43)	-0.63^†^ (0.34)	-0.82 (0.62)	-2.00^†^ (1.12)
No or lowest qualification	-1.63^†^ (0.86)	-0.64 (1.58)	-2.94** (0.98)	-0.19 (0.36)	0.39 (0.33)	-0.25 (0.51)	-0.47 (0.73)
Intermediary secondary qualification	-1.12 (1.16)	-0.15 (2.17)	-2.14* (1.04)	0.04 (0.33)	0.36 (0.32)	-0.17 (0.53)	-0.00 (0.69)
Indirect effect via	0.25 (0.28)	-0.20 (0.33)	-0.01 (0.21)	0.39* (0.15)	0.51** (0.16)	0.30* (0.15)	0.11 (0.19)
internal political efficacy^e^	0.54** (0.20)	0.54*** (0.20)	0.58** (0.19)	0.56*** (0.14)	0.56*** (0.14)	0.56*** (0.14)	0.56*** (0.14)
*Ṟ*^2^(political participation)	.407	.412	.262	.168	.185	.076	.139
*R*^2^(internal political efficacy)	.215	.215	.197	.206	.206	.203	.203

*Note*. Unstandardized regression weights (standard errors in parentheses).

^a^Pre-election sample with *n* = 1,146. ^b^Post-election sample with *n* = 1,156. ^c^*n* = 2,291. ^d^*n* = 2,336. ^e^Indirect effects above (upper row); direct effects of political knowledge on internal political efficacy below (lower row).

^†^*p* < .101. *p < .05. **p < .01. ***p < .001.

Although our results support previous research which, in the German context, suggested that political knowledge may have an indirect effect on conventional political participation, mediated by political efficacy (Reichert, 2013), they are not in line with the assumption that internal efficacy should always facilitate actual behaviour (Ajzen, 1991). Yet it was outlined earlier that we may expect that internal efficacy may have a significant impact on behavioural intentions independent of its influence on actual behaviour, in particular given the time interval between each survey. Therefore, the following deals with the question whether the impact of internal political efficacy—though it did not predict voting and unconventional participation (the latter only applies to the 2009 analysis) directly—was perhaps mediated by behavioural intentions.

This seems plausible, because the intention to participate in respective political activities increased the likelihood that a person would actually participate (although for unconventional participation, it was not significant at the five per cent level of significance). It is noticed again that the common requirement that there be a significant zero-order correlation between predictor and outcome variable “severely reduces power to detect mediation, especially in the case of complete mediation (i.e., direct effect is zero)” (MacKinnon et al., 2007, p. 601). This even more so if the mediation process becomes less proximal and/or the impact of a predictor variable is mediated in several sequences (Shrout & Bolger, 2002), which may be the case in our longitudinal analysis owing to the time between the measurement of political knowledge and the occurrence of actual political participation. Following these thoughts, sequential mediation might be a plausible alternative mechanism of how political knowledge translated into political action. Therefore, the following examines whether behavioural intentions worked as a second mediator, such that political knowledge may have increased internal political efficacy, which promoted citizens’ willingness to participate in politics, and thereby affected political participation on a sequentially mediated path.

#### The Mediating Influence of Behavioural Intentions

##### Preliminary Cross-Sectional Analyses

Prior to sequentially mediated regression analyses, cross-sectional analyses were performed to examine whether political efficacy predicted behavioural intentions and, hence, mediated the influence of political knowledge. Table 2 shows the respective regression coefficients, with intentions to political action as the main criteria, whereas internal political efficacy was regarded as a mediator of political knowledge and, therefore, was an intermediate criterion.

**Table 2:** Cross-Sectional Mediated Regression Analyses (Criteria: Intentions to Participate)

Predictor	Voting (Pre-Election Sample)^a^	Conventional Participation^b^	Unconventional Participation^b^
Political knowledge	0.14 (0.22)	0.25 (0.18)	0.36* (0.16)
Internal political efficacy	0.15*** (0.03)	0.38*** (0.03)	0.43*** (0.03)
Age	0.01*** (0.00)	-0.01*** (0.00)	-0.02*** (0.00)
Gender	-0.07 (0.05)	-0.08 (0.06)	-0.04 (0.05)
Pre-/Post-election		-0.15** (0.05)	-0.09^†^ (0.05)
Income	0.01 (0.01)	0.02^†^ (0.01)	0.00 (0.01)
Region of residence	-0.23*** (0.06)	-0.21*** (0.05)	0.08^†^ (0.05)
No or lowest qualification	-0.16* (0.06)	-0.25** (0.08)	-0.42*** (0.06)
Intermediary secondary qualification	-0.10 (0.06)	-0.16* (0.07)	-0.28*** (0.06)
Indirect effect via	0.07* (0.03)	0.21*** (0.05)	0.24*** (0.06)
internal political efficacy^c^	0.51* (0.19)	0.55*** (0.14)	0.55*** (0.14)
*R*^2^(intention to participate)	.077	.144	.259
*R*^2^(internal political efficacy)	.216	.203	.203

*Note*. Unstandardized regression weights (standard errors in parentheses).

^a^*n* = 1,195. ^b^*n* = 2,353. ^c^Indirect effects above (upper row); direct effects of political knowledge on internal political efficacy below (lower row).

^†^*p* < .101. *p < .05. **p < .01. ***p < .001.

We find that political knowledge was barely a significant predictor of intentions to participate politically if we controlled for multiple predictors, as it only increased the intention to participate in unconventional political behaviour. In contrast, and as suggested by TPB, internal political efficacy increased behavioural intentions throughout. These results support the assumption that political knowledge translates into internal political efficacy and may affect political participation only indirectly—perhaps fully mediated by efficacy and behavioural intentions.

##### Voting

**Figure 1:**
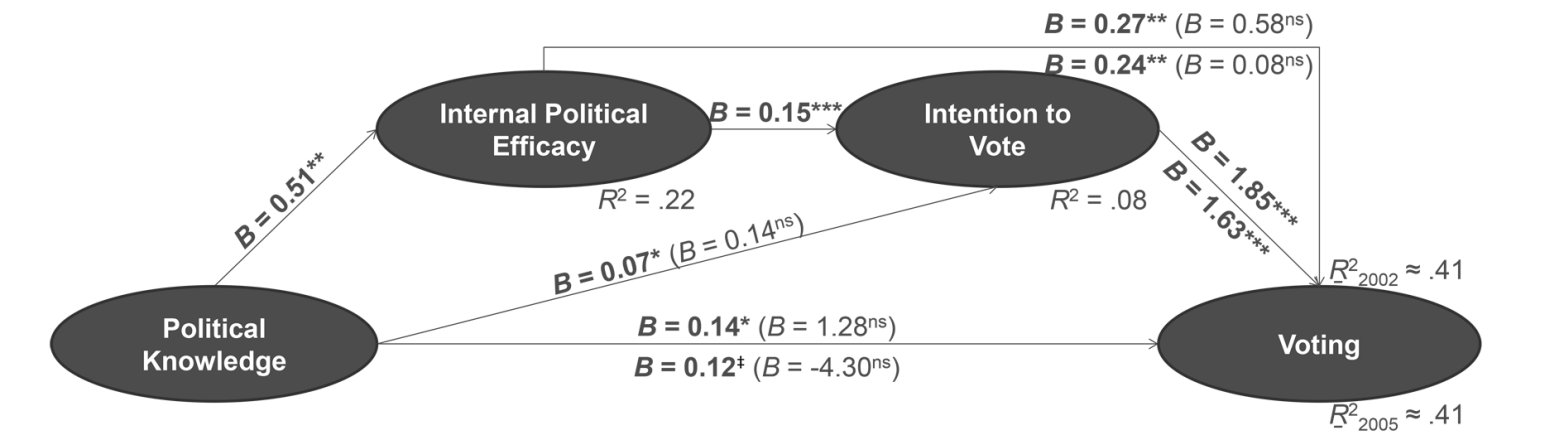
Prediction of voting in the federal election by political knowledge, sequentially mediated through internal political efficacy and intention to vote (effects on voting in 2002 above lines; effects on voting in 2005 below lines; pre-election sample with *n* = 1,195). *Note.* Unstandardized regression weights; direct effects in mediated regressions in parentheses. ^‡^*p* < .051. *p < .05. **p < .01. ***p < .001. ns = not significant.

Figure 1 indeed shows a sequentially mediated effect in the pre-election sample. Political knowledge raised internal political efficacy, which increased the willingness to vote in the upcoming election, and finally this intention predicted actual voting. Internal political efficacy did not directly affect voting behaviour, but it had an indirect influence through the conscious decision actually to vote. This pattern emerged for both voting in the 2002 as well as voting in the 2005 federal election.

##### Conventional Political Participation

**Figure 2:**
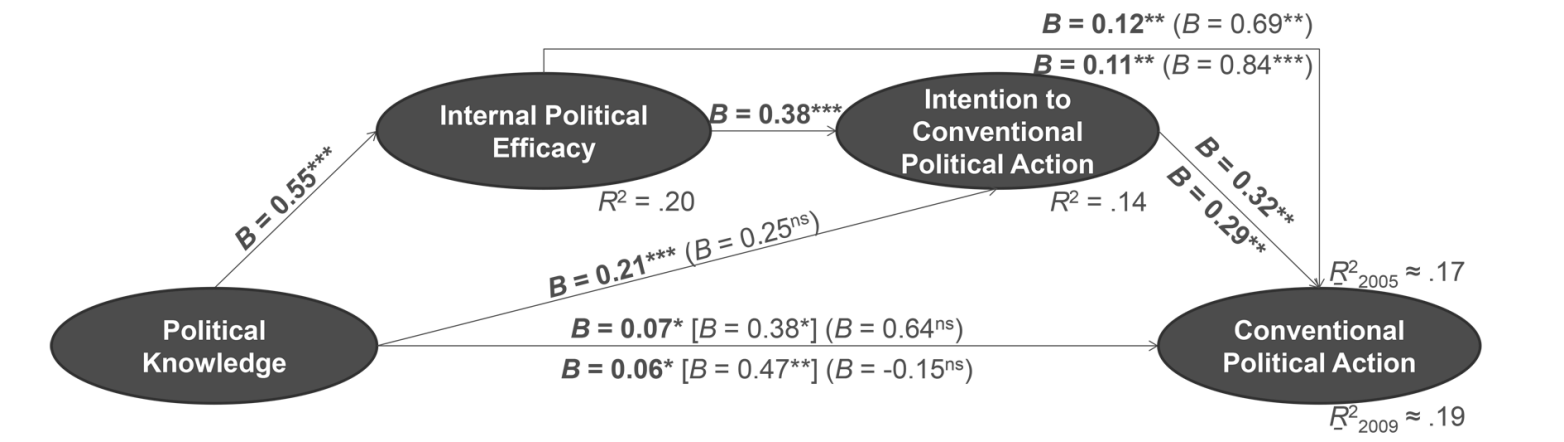
Prediction of conventional political participation by political knowledge, sequentially mediated through internal political efficacy and intention to participate in conventional political action (effects on conventional participation between 2002 and 2005 above lines; effects on conventional participation between 2005 and 2009 below lines; *n* = 2,353). *Note.* Unstandardized regression weights; direct effects in mediated regressions in round parentheses; indirect effect of political knowledge by internal political efficacy on actual behaviour in squared parentheses. *p < .05. **p < .01. ***p < .001. ns = not significant.

This time looking at longitudinal effects, Figure 2 confirms that internal political efficacy had an indirect effect on actual behaviour *in addition* to its direct influence, which was mediated by the intention to participate. That is, political knowledge affected the intention to participate in conventional action in the future indirectly through internal efficacy, and it was sequentially mediated by both efficacy and willingness to act. It comes without surprise that political efficacy also mediated the impact of political knowledge on conventional participation in a “direct” step. This pattern is identical for conventional political participation measured in 2009 (Figure 2).

##### Unconventional Political Participation

**Figure 3:**
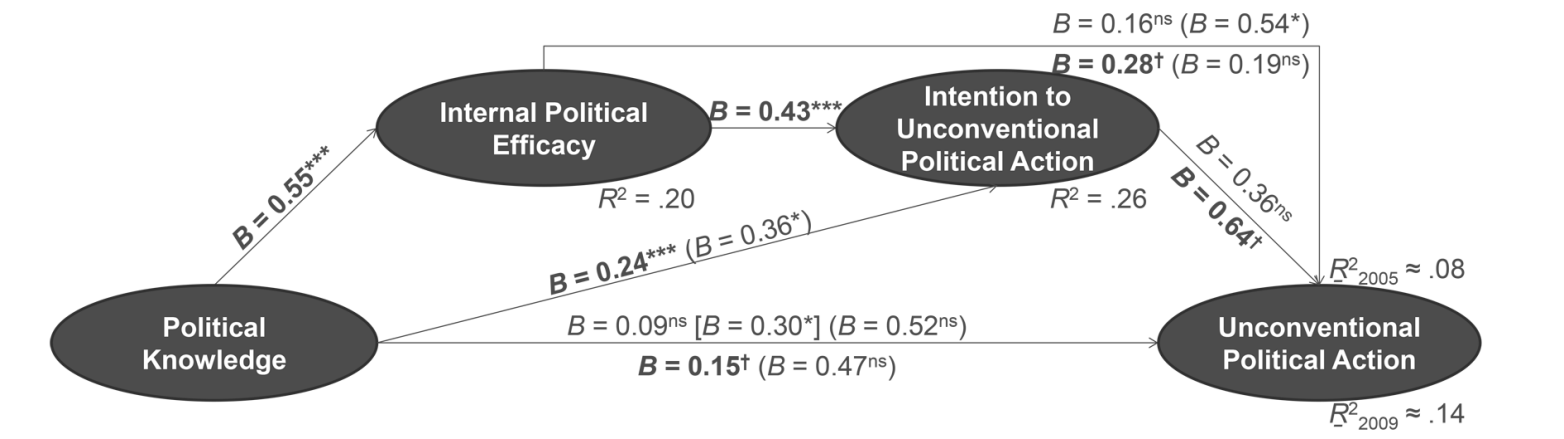
Prediction of unconventional political participation by political knowledge, sequentially mediated through internal political efficacy and intention to participate in unconventional political action (effects on unconventional participation between 2002 and 2005 above lines; effects on unconventional participation between 2005 and 2009 below lines; *n* = 2,353). *Note.* Unstandardized regression weights; direct effects in mediated regressions in round parentheses; indirect effect of political knowledge by intention to participate on actual behaviour in squared parentheses. ^†^*p* < .101. *p < .05. **p < .01. ***p < .001. ns = not significant.

When we look at Figure 3 and unconventional participation, we see that political knowledge elevated the willingness to participate in unconventional political participation directly. Yet we also discover an additional indirect effect which was mediated by internal political efficacy, i.e. political knowledge increased the intention to participate in unconventional political action in two ways: indirectly by promoting an individual’s internal political efficacy which in turn affected their willingness to participate; and it was also a direct facilitator of the willingness to unconventional participation. Consequently, political knowledge did not necessarily have to translate to internal political efficacy in order to trigger unconventional participation.

However, the behavioural intention was only a marginally significant predictor of actual behaviour in 2009, but it was no significant precursor of unconventional participation in 2005. As a result, the mediated paths to actual participation differed in both years. In 2005, our analysis yielded no statistical evidence for a sequential mediation. Instead, political knowledge was mediated by internal political efficacy in one step only. For unconventional participation between 2005 and 2009, however, a marginally significant sequential mediation occurred (Figure 3): Political knowledge boosted internal political efficacy, thereby it increased the intention to participate in unconventional political behaviour indirectly, which in turn raised the chance that a citizen would participate unconventionally. As efficacy was no significant predictor of unconventional participation in the latter analysis, the one-step mediated path that was identified in the 2005 analysis did not occur in 2009.

## Discussion

This paper contributes to the understanding of how political knowledge translates into political action through the application of the Theory of Planned Behaviour (TPB) and by focusing on internal political efficacy as its mediator. We expected that political knowledge would be fully mediated by internal political efficacy with respect to conventional political participation, though it would retain some impact on voting and unconventional political behaviour (H1, H2). We found evidence which underlines the significance of internal political efficacy, but political knowledge had no direct influence on actual participation. However, as it raised the levels of internal political efficacy, political knowledge was fully mediated and turned into action in an indirect way.

This was not a simple translation though: Internal political efficacy contributed to the intention to participate in all kinds of political action, while efficacy only predicted increases in the likelihood to participate in conventional political behaviour in a direct way. As we had hypothesized, the impact of internal political efficacy was mediated by behavioural intentions (H3), but only in the prediction of conventional political participation did efficacy consistently retain an additional direct effect on actual behaviour. A citizen’s internal political efficacy seemed to be more important with regard to conventional political participation compared with their willingness to participate, but it was far from clearly being much more important than the impact of the latter (H4). In those cases when efficacy predicted behavioural intentions and these affected actual participation in multiple analyses, we nevertheless found sequentially mediated effects, that is, political knowledge raised internal political efficacy; thereby it contributed to the willingness to participate politically, which in turn increased the likelihood that a citizen would participate in politics.

The present research adds to few studies that employ an action theoretic model to examine the role of accurate knowledge in the prediction of behaviour. It specifically shows that political knowledge is rather subsidiary compared with perceived behavioural control (Ajzen et al., 2011), meaning that internal political efficacy is more important in the prediction of political behaviour. Adding to that, political knowledge affected political participation through internal political efficacy, and it primarily affected behavioural intentions (compare also Dalrymple et al., 2013; Polonsky et al., 2013). Although accurate political knowledge may not always be necessary, nor is it sufficient, to predict behaviour (Ajzen et al., 2011), it contributes to decisions that conform with an individual’s preferences and political attitudes (Galston, 2001), at least indirectly. Also, it is a precursor of more proximal beliefs and personal preferences that closely relate to actual participation (e.g., Polonsky et al., 2013).

Previous research has discussed such a mediation in the political realm (Delli Carpini & Keeter, 1996; Jung et al., 2011; Reichert, 2013), while others found the effects of efficacy on knowledge to be stronger than vice versa (Vetter & Maier, 2005). They did not test the indirect path on political participation in mediated regression analysis, however. The present study went one step further and tested for the mediated effect of political knowledge, as well as a sequential mediation via both efficacy and intended participation. Yet direct effects of efficacy on actual participation were rarely found when we controlled for willingness to participate, which is surprising given that TPB suggests simultaneous direct effects of both efficacy and behavioural intentions on actual behaviour (Ajzen, 1991, 2012).

Admittedly, this article examined only one part of TPB. However, we found direct positive influences of behavioural intentions *and* internal political efficacy in the prediction of conventional political participation, in addition to mediated effects through behavioural intentions. Here internal political efficacy rather outweighed the impact of behavioural intentions. Those direct effects on conventional political participation above and beyond intentions are congruent with previous research (Reichert, 2013), which shows a higher significance of internal political efficacy with respect to conventional compared to other forms of political participation. Internal political efficacy might thus be more relevant for initiating party-political activities that require a strong and continuous commitment compared to one-off or issue-based political activities that focus on just one specific political goal or act.

### Limitations

In sum, we found evidence in support of our hypotheses, although the effect sizes were rather small and a few mixed results also require us to address some constraints of this study. First, the intention to participate in unconventional political action did not (or barely) increase the likelihood actually to participate unconventionally. This might be due to one limitation stemming from the measures on unconventional political participation that we could use in our analysis: One constraint using the GLES long-term panel was the high percentage of respondents who actually voted in federal elections, and that only one item for unconventional behaviour was available, which yielded a very small number of people who participated unconventionally. This latter item certainly reflects issue-related participation, yet many other forms of unconventional participation exist, such as participating in a protest march or boycotting products for political reasons. Still, it is surprising that the expected relationships appeared for voting and party-related political participation but not for unconventional behaviour. Further research is needed to understand whether the (almost) absence of the expected mediated relationships generally applies to unconventional participation. This could indeed be the case though, assuming that unconventional political participation is less continuous than or perhaps not as much a consistent habit as participation in more institutionalized ways. That is, a citizen’s actual participation in unconventional activities might be less predictable, as specific political issues and, hence, opportunities to issue-based participation may arise on rather short notice.

Furthermore, the samples were also affected by panel attrition, yet this is one of the problems in longitudinal research. In any event, both the sampling strategy and the changes in measurement are limitations of this study, just as we need to be aware that political knowledge and the motivation to participate in surveys are confounded (Prior & Lupia, 2008). However, the dataset used here is one of few nationwide panel studies, and to the author’s knowledge the only longitudinal database which also provides all measures that were required to test the mediation hypotheses (i.e. a comprehensive political knowledge measure, internal political efficacy, behavioural intentions and self-reported actual participation in different kinds of political participation).

Lastly, a minor point refers to the measurement of political knowledge. The employed measure reflected knowledge which was relevant in the (upcoming) federal election(s). Whereas Johann (2012) found significant effects of political knowledge not merely on voting but on different political activities even when he controlled for internal political efficacy, in our analyses the only direct influence of political knowledge was on expected participation in unconventional activities in the future. However, the results for conventional political participation are similar to research that used a broader knowledge measure (Reichert, 2013), and are quite aligned for voting and conventional participation. Therefore, the present study provides significant insights despite of the rather narrow content captured by its knowledge items. Yet we need to address this as a minor restriction with respect to the generalizability of our findings.

### Conclusion

In conclusion, the present analysis provides indication that internal political efficacy increases intentions to participate politically. It is well known that behavioural intentions are important preconditions of actual behaviour (Ajzen, 2012; Westholm, Montero, & van Deth, 2007), far from being perfectly correlated though (Fishbein & Ajzen, 1981; Gaiser & de Rijke, 2010). We applied longitudinal analyses using *actual* political participation. Thus, we could corroborate previous research (Reichert, 2013), in particular with regard to conventional political participation. In essence, the results of the present study seem reliable, but further research, both quasi-experimental and survey research, is needed to understand the different patterns for voting (sequentially mediated impact) and party-related (both sequentially and “directly” mediated influences) versus unconventional political participation (mixed results) in detail. In addition, researchers need to consider various unconventional political activities, as the measure in the present study was somewhat problematic (see above).

The findings furthermore raise questions for examination in future research. That research may focus on potentially differential effects of knowledge about political figures versus knowledge about polity on various forms of political participation (Johann, 2012; Reichert, 2013). Here media exposure may play a significant role, as policy-specific knowledge is highly influenced by the breadth of media coverage and the prominence of a certain issue (Barabas & Jerit, 2009), though it also matters how the actual information is made available (i.e. “digestible”) (Jerit, Barabas, & Bolsen, 2006) and by whom (Munger, Egan, Nagler, Ronen, & Tucker, 2015). Polity-related knowledge may be harder to acquire through the media, however. In addition it would be useful to consider profound competences such as political reasoning or capacities to act, in addition to accurate political knowledge, when assessing the mediating effect of political efficacy.

Most important though will be replication studies using cross-national data to examine how general or specific these relationships are. Only then will we be able precisely to answer the questions of how, when, and which facets of political knowledge affect certain kinds of political participation, and to whom those findings apply. The present study focused on Germany; however, it showed that action theory provides a valid approach to the study of participation in political realm (Diener, Noack, & Gniewosz, 2011). In conclusion, this research suggests that political knowledge is far from being unimportant, though it has to translate into political efficacy to trigger political participation. Strengthening political knowledge and internal political efficacy to increase the levels of political attentiveness and participation (Pasek, Feldman, Romer, & Jamieson, 2008) are significant challenges for civics educators to bring about politically engaged and democratically participating citizenries.

## Appendix

**Table A1:** Means and Standard Deviations of the Independent, Mediator, and Dependent Variables

Variable	Mean (Standard Deviation) / Frequency
Political knowledge 2002	0.31 (0.15)
Internal political efficacy 2002	2.24 (0.93)
Intention to vote (2002, pre-election)	3.67 (0.84)
Intention to conventional participation (2002)	1.75 (1.34)
Intention to unconventional participation (2002)	1.90 (1.23)
Voting 2002 (pre-election)	96%
Voting 2005 (pre-election)	96%
Voting 2009 (post-election)	93%
Conventional participation 2005	11%
Conventional participation 2009	20%
Unconventional participation 2005	3%
Unconventional participation 2009	4%

**Table A2:** Bivariate Correlations Between Political Participation and Political Knowledge and Efficacy

Kind of Political Participation	Political Knowledge	Internal Political Efficacy
Intention to vote (2002, pre-election sample)	.05^†^	.20***
Voting (2002, pre-election sample)	.07	.13**
Voting (2005, pre-election sample)	.04	.16**
Voting (2009, post-election sample)	.11^†^	.10
Intention to conventional action (2002)	.07***	.31***
Conventional participation (2005)	.04	.18***
Conventional Participation (2009)	.06	.23***
Intention to unconventional action (2002)	.11***	.40***
Unconventional participation (2005)	.02	.12***
Unconventional Participation (2009)	.07	.10**

*Note.* Political knowledge, internal political efficacy and behavioural intentions measured in 2002 (weighted *n* between 291 and 3,016).

^†^*p* < .101. **p* < .05. ***p* < .01. ****p* < .001.
